# Dimensions of the Hamilton Depression Rating Scale Correlate with Impulsivity and Personality Traits among Youth Patients with Depression

**DOI:** 10.3390/jcm12051744

**Published:** 2023-02-22

**Authors:** Aleksandra Rajewska-Rager, Monika Dmitrzak-Weglarz, Natalia Lepczynska, Pawel Kapelski, Joanna Pawlak, Aleksandra Szczepankiewicz, Marcin Wilczynski, Maria Skibinska

**Affiliations:** 1 Department of Psychiatric Genetics, Poznan University of Medical Sciences, Rokietnicka 8, 60-806 Poznan, Poland; 2 Department of Child and Adolescent Psychiatry, Karol Jonscher Clinical Hospital, Poznan University of Medical Sciences, Szpitalna 27/33 St, 60-572 Poznan, Poland; 3 Molecular and Cell Biology Unit, Poznan University of Medical Sciences, 60-572 Poznan, Poland

**Keywords:** depression, factor analysis, Hamilton Depression Rating Scale (HDRS-17), affective dimensions, adolescents, young adults

## Abstract

The heterogeneity of symptoms in young patients with major depression disorder makes it difficult to properly identify and diagnose. Therefore, the appropriate evaluation of mood symptoms is important in early intervention. The aim of this study was to (a) establish dimensions of the Hamilton Depression Rating Scale (HDRS-17) in adolescents and young adults and (b) perform correlations between the identified dimensions and psychological variables (impulsivity, personality traits). This study enrolled 52 young patients with major depression disorder (MDD). The severity of the depressive symptoms was established using the HDRS-17. The factor structure of the scale was studied using the principal component analysis (PCA) with varimax rotation. The patients completed the self-reported Barratt Impulsiveness Scale (BIS-11) and Temperament and Character Inventory (TCI). The three dimensions of the HDRS-17 identified as core in adolescent and young patients with MDD were (1) psychic depression/motor retardation, (2) disturbed thinking, and (3) sleep disturbances/anxiety. In our study, dimension 1 correlated with reward dependence and cooperativeness; dimension 2 correlated with non-planning impulsivity, harm avoidance, and self-directedness; and dimension 3 correlated with reward dependence. Conclusions: Our study supports the previous findings, which indicate that a certain set of clinical features (including the HDRS-17 dimensions, not only total score) may represent a vulnerability pattern that characterizes patients with depression.

## 1. Introduction

Major depression disorder (MDD) is a heterogeneous disorder in many aspects: a variety of symptoms, the onset of disease (early-onset, late-onset), the course of the illness (single episode vs. recurrent), comorbidities, and multifactorial etiology (biochemical, immunological, genetic) [1,2,3]. The complexity of depression makes it difficult to indicate the main etiological factor. Within several pathophysiological concepts, recent studies focused on the biomarkers of brain plasticity and the functional connectivity between brain regions using neuroimaging and electroencephalography [4,5]. Neurobiological research in MDD shows altered cortical activity, e.g., loss of dendritic complexity in the hippocampus and prefrontal cortex and neurotrophic abnormalities [6,7,8]. Impaired cellular resilience and loss of neuroplasticity are indicated to be responsible for these changes [8]. Dysfunctional cognitive processes, as observed in patients with depression, are also associated with the altered function of specific brain regions, mainly in the prefrontal areas and cingulate cortex, amygdala, and hippocampus [9]. Neuroendocrine tests in combination with genotyping and stress sensitivity are another research direction due to the confirmed dysregulation of the hypothalamus–pituitary–adrenal (HPA) axis and increased systemic inflammation [10,11]. Multivariate risk factors as well as psychological factors include personality traits and impulsivity, especially in the early presentation of the illness [12]. Depression in youth leads to significant educational and social impairment, which exacerbates loneliness, and is a significant risk factor in suicide [13,14,15]. Research shows that 60–70% of adult patients experience their first affective symptoms before the age of 18 [16,17,18]. The primary presenting symptoms in youth are often unexplained physical symptoms, mood reactivity, eating symptoms, anxiety, refusal to attend school, decreased school performance, and/or behavioral problems [19,20,21,22]. Due to the heterogeneity related to different symptom constellations meeting the DSM (Diagnostic and Statistical Manual of Mental Disorders) diagnostic criteria for major depression, patients with the same diagnosis can experience various clinical manifestations of their illness [3,23,24]. Research efforts should aim to elucidate the heterogeneity of depression in this age.

As discussed above, mood symptoms in young patients are more challenging to assess than the symptoms manifested by adults. The profiling of symptom patterns is valuable for risk assessment and might be a step towards developing tailored intervention for young patients. The principal component analysis (PCA) is an approach that helps identify significant components or latent dimensions within a heterogeneous diagnostic construct [25]. A dimensional model of mood symptoms may be more effective in screening and assessing depressive disorders in youths. However, in contrast to adult depression, there has been limited investigation of a dimensional symptom perspective or the psychological factors involved in mood regulation at an early age [26]. Factor (or related, e.g., PCA) analyses have been performed to define the depressive symptom clusters within the HDRS scale in adult patients with mood disorders [27,28,29]. The available studies show that “symptom clusters” derived using the factor analysis of the Hamilton Depression Rating Scale (HDRS) might be more informative regarding clinical characteristics than the total HDRS scores [30]. The number of identified factors ranged between two and eight [28,31]. In general, factor analyses identify clusters of “depressive factor”, ‘anxiety factor’, “somatic or neurovegetative factor”, “psychomotor retardation”, and ‘sleep disturbance factor’ [27,28,32,33].

The diagnosis of MDD, especially in youths, should be characterized by the multitude of pathophysiological components involved. MDD, as a multifactorial disorder, is associated with stress-related pathophysiological components. Impulsivity and specific personality traits are the crucial psychological components that influence patient reaction and stress adaptation [34,35]. Among the various personality assessment scales, the Temperament and Character Inventory (TCI), the psychobiological personality concept developed by Cloninger, is the most commonly used in clinical research [36]. Cloninger’s theory implies a relation between individual personality dimensions with neurotransmitter systems, whilst character dimensions are influenced more by social learning than genetic factors. Earlier research on adolescent patients found significant and positive correlations between depressive symptoms and different impulsivity subtypes [37,38].

We conducted a principal component analysis (PCA) based on psychopathological characteristics assessed in young patients with depression. We included clinical depressive symptoms as well as features such as impulsivity and personality traits as potential psychological markers of depression, which may represent an underlying psychopathological vulnerability towards the development of MDD. Classifying psychopathology based on dimensions and identifying reliable predictors of illness may allow more specific interventions [25,39]. Potentially, additional homogeneous dimensions within a broad psychopathological assessment in youth patients with depression would facilitate diagnosis.

In our study, we hypothesized that, due to various clinical symptom presentations in young patients with depression, it is possible to identify specific sets of symptoms that correspond to the characteristics associated with depression for patients at a young age with a correlation to psychological factors, and that these factors will differ from those observed in adults. This study aimed to (1) establish different dimensions of the HDRS-17 in adolescents and young adults and (2) establish correlations between the identified dimensions and psychological variables (such as impulsivity and personality traits).

To the best of our knowledge, no previous study has evaluated depressive clusters with psychological dimensions in young patients with mood disorders. A clinical approach that considers psychopathology as multiple dimensions could help to individualize clinical evaluation at a young age and provide a more precise diagnosis. Therefore, to better understand depression symptomatology, a detailed symptom-level analysis in adolescence is a promising direction to capture all features of depression as it relates to young people [40]. Further, identifying specific symptomatic profiles or depression clusters might allow for a better selection of treatment interventions.

## 2. Materials and Methods

### 2.1. Participants

In this study, we enrolled 52 Caucasians, adolescents and young adults (aged 14–24 years) of Polish origin, who were diagnosed with major depressive disorder (MDD). Patients were recruited from both inpatients and outpatients (Department of Child and Adolescent Psychiatry and Adult Psychiatry Department of Poznan University of Medical Sciences). This study was performed following the Declaration of Helsinki, and the Ethics Committee approved the study protocol (no. 362/11). Sociodemographic and clinical data were collected from all participants. The clinical information, including medical and psychiatric history, was synthesized and archived in computerized records. All study participants or their legal guardians gave written informed consent to participate in the study. A diagnosis of major depression was established according to the ICD-10 and DSM-IV criteria by two independent trained psychiatrists. Depending on the age of the participant, we used either the Kiddie Schedule for Affective Disorders and Schizophrenia—Present and Lifetime Version (KSADS-PL) dedicated to children and adolescents [41] or the Structured Clinical Interview for DSM-IV (SCID) for young adults [42]. The inclusion criteria included Polish origin Caucasian, young age between 14 and 24 years, major depression diagnosis, inpatients or outpatients, and no comorbidity in axis I or axis II disorder. The exclusion criteria included the presence of severe medical or neurological illness, intellectual disability, pervasive developmental disorder, and pregnancy.

### 2.2. Clinical Assessment

The Kiddie Schedule for Affective Disorders and Schizophrenia–Present and Lifetime Version (KSADS-PL) is the most commonly used semi-structured interview, which helps to achieve a better clinical evaluation for each symptom and facilitates early diagnosis of affective and other mental disorders corresponding to the DSM criteria [23]. This interview is designed to collect detailed information about mental symptoms from the child or adolescent and their parents or other informants. The KSADS-PL has six components, containing an introductory interview, general patient data, as well as a detailed screening section of the main symptoms of each disorder; it takes approximately one hour to complete. The majority of items in the KSADS-PL are scored using a 0–3 point rating scale: Scores of 0—no information available, scores of 1—symptom is not present, scores of 2—sub-threshold presentation, and scores of 3—threshold presentation of symptoms [41].

The Structured Clinical Interview for the Diagnostic and Statistical Manual of Mental Disorders is a semi-structured interview dedicated to the assessment of adult patients. This diagnostic instrument helps to determine reliable adult psychiatric diagnoses according to the DSM criteria. The interview has an overview section with basic demographic information and the chief complaint, and then a descriptive part with open-ended questioning and questions related to diagnostic criteria, which are asked in a closed-ended (i.e., yes/no) fashion. This interview assesses both current and lifetime diagnoses, and the questions are grouped by diagnosis and criteria; it takes approximately one hour to complete [42].

To evaluate the depressive symptoms, the clinician-rated Hamilton Depression Rating Scale (HDRS-17) was used, which considers 17 items scored between 0 and 4 points [43]. The Hamilton Rating Scale is designed to measure the severity of depressive symptoms, such as depressed mood, guilt, suicidality, insomnia (early, middle, and late), anhedonia, psychomotor retardation, agitation, psychological and somatic anxiety, somatic symptoms, genital symptoms, hypochondriasis, insight into condition, weight loss, diurnal variation, derealization, paranoid symptoms, and obsessional and compulsive symptoms. Each item is scored from 0 to 3 depending on the severity of the depressive symptoms with cut-off points for depressed mood ≥8 points. The scoring ranges are 0–7 no depressive symptoms, 8–16 mild depression, 17–23 moderate depression, and over 24 severe depression. Participants also completed the self-reporting Barratt Impulsiveness Scale (BIS-11), scored on a four-point Likert scale (higher total scores indicate a higher impulsivity trait). The BIS scale is the most widely used measure of impulsive personality traits. It assesses total impulsiveness and first-range symptoms: non-planning, and motor and attentional impulsiveness [44,45]. In our study, to assess personality traits, we used the Temperament and Character Inventory (TCI)—a 240-item self-reporting inventory—to assess the personality dimensions [36]. The TCI was developed by C. Robert Cloninger and is based on a psychobiological model that attempts to provide and explain the underlying causes of individual differences in personality traits. Responses in the scale are based on dichotomous answer items (true/false). There was no time limit to complete the questionnaire. The inventory consists of seven personality dimensions: four temperaments (harm avoidance HA, reward dependence RD, novelty-seeking NS, persistence P) and three character dimensions (self-directedness SD, cooperativeness C, self-transcendence ST).

### 2.3. The Statistical Analyses

For the estimation of the HDRS-17 dimensions, the principal component analysis with varimax rotation was performed. The threshold for inclusion of an item in one factor was 0.43. This threshold allowed for the grouping of each HDRS-17 item exclusively in one dimension, leaving only two unassigned items. Lowering the threshold, the individual items were grouped in more than one dimension. Increasing the threshold, more items were left unassigned. Subsequently, we estimated the dimension scores for each patient by adding the values of the items included in each factor. The normality of the data was checked using the Lilliefors test. The distribution of dimension 1; dimension 2; TCI: harm-avoidance, persistence, self-transcendence; and BIS-11: attentional and non-planning were skewed. Therefore, we performed a non-parametric Spearman’s correlation. The Spearman’s correlation was performed on the HDRS-17 dimensions with the total BIS-11 score; the second-order BIS-11 factors: non-planning, and motor and attentional impulsiveness; and TCI personality dimensions: harm avoidance, reward dependence, novelty-seeking, persistence, self-directedness, cooperativeness, and self-transcendence.

The influence of the covariates, age, and gender on the HDRS-17 dimensions was analyzed using the analysis of covariance (ANCOVA) with a post-hoc Tuckey test and the Levene test for equality of variance The significance level was set at *p* = 0.05. The statistical analyses were performed using Statistica v.13 program (StatSoft, Poland). The post-hoc power of the correlation analysis was estimated using an online calculator (https://sample-size.net/correlation-sample-size/, accessed on 18 January 2022).

## 3. Results

### 3.1. Demographic and Clinical Variables

The mean age of the participants was 18.67 (±3.54) years, and 75% were female. The mean HDRS score was 19.37 (±5.30). The participants were significantly more often inpatients than outpatients: 69% of the study group was inpatient. A total of 40% of the patients were drug-free. The patients who received pharmacological treatment had selective serotonin reuptake inhibitors (37%), selective norepinephrine reuptake inhibitors (6%), tricyclic antidepressants (4%), and others, e.g., anxiolytics, sleeping pills (14%) (Table 1).

The obtained mean BIS-11 and TCI dimensions from the study group are shown in Table 2.

### 3.2. Principal Component Analysis (PCA)

In our study, we used the principal component analysis (PCA) with a varimax rotation and item loadings of 0.4 or greater. We extracted three dimensions in adolescents and young adults with depression. Dimension 1 was defined by depressed mood (item 1), work and activities (item 7), retardation (item 8), psychic anxiety (item 10), somatic symptoms (item 13), genital symptoms (item 14), and loss of weight (item 16). As the items with higher loadings on this factor were depression and retardation, we identified dimension 1 as ‘psychic depression and motor retardation.’ Dimension 2 consisted of the following items: feelings of guilt (item 2), suicide (item 3), and somatic gastro-intestinal symptoms (item 12). The first two symptoms were identified as the ‘disturbed thinking’ dimension because of the higher significance. Dimension 3, ‘ Sleep Disturbances and Anxiety,’ covered sleeping disturbance and somatic anxiety symptoms (items assessing early (4), middle (5), and late insomnia (6), agitation (9), and somatic anxiety (11)). The unassigned items were hypochondriasis (15) and insight (17). The results of the Hamilton Depression Rating Scale (HDRS-17) principal component analysis are shown in Table 3.

### 3.3. Spearman’s Correlation of the HDRS-17 Dimensions with BIS-11 and TCI

In the correlation analysis using the Barratt Impulsiveness Scale (BIS-11) and Temperament and Character Inventory (TCI), we found a positive correlation between non-planning impulsivity (*p* = 0.02, power 0.61) and harm avoidance (HA) (*p* = 0.01, power 0.71) with dimension 2 of the HDRS. The temperament dimension reward dependence (RD) was positively correlated with dimension 1 (*p* = 0.04, power 0.54) and dimension 3 (*p* = 0.02, power 0.62). Reward dependence (RD) and self-directedness (SD) were negatively correlated with dimension 2 (*p* = 0.03, power 0.58 and 0.01, power 0.74; respectively). Cooperativeness (C) was positively correlated with dimension 1 (*p* = 0.01, power 0.69).

Results of the correlation analysis are presented in Table 4.

### 3.4. The Effect of Age and Gender on the HDRS-17 Dimensions

The ANCOVA analysis detected a significant effect of age on all three HDRS-17 domains: the first (F = 20.18, *p* = 0.00004), the second (F = 14.48, *p* = 0.0004), and the third (F = 11.22, *p* = 0.001). Female patients had higher scores of dimension 2 compared to males (F = 11.2, *p* = 0.001).

## 4. Discussion

To our knowledge, this is the first study to evaluate dimensions for the HDRS-17 items alongside a correlation with psychosocial factors in adolescents and young adults. The first aim of the present study was to identify the dimensions of the HDRS-17 within adolescents and young adult MDD samples. Second, we performed correlations between the identified dimensions and psychological variables (impulsivity, personality traits). The principal component analysis revealed three independent factors. Psychic depression and psychomotor retardation were extracted as the first dimension (characterized mainly by depressed mood, decreased activities, motor retardation, and somatic and genital symptoms). Dimension 2 was labeled the disturbed thinking factor (characterized by a feeling of guilt and suicidality). Dimension 3 was labeled as sleep disturbance/anxiety (which included mainly insomnia and agitation).

### 4.1. Principal Component Analysis of the HDRS-17

Previous studies on adults with depression identified factors in the HDRS scale defined mostly by items covering the core symptoms of depression with differences among the studies regarding the variables included in the separate factors with significance, as estimated by the size of factor loadings [43,46,47]. Bagby et al. (2004) identified 15 studies on adult patients that analyzed the HDRS-17 factors; the number of factors ranged between two and eight. In most studies, insomnia items appeared consistently in the same ‘sleep disturbance’ factor. In six studies, depressed mood, guilt, and suicide were loaded together in the same factor of ‘general depression’. Another seven data sets on the same factor included the combination of depressed mood, suicide, and psychic anxiety. They also identified an ‘anxiety factor’ in six studies, which consisted of agitation, psychic anxiety, and somatic anxiety [28]. By contrast, in the study by Wade et al. (2020), the HDRS-17 items were loaded in the three-factor model. The first factor captured the core aspects of depression: work and interests, weight loss, psychomotor retardation, and depressed mood. The second factor largely included the items reflecting somatic gastro-intestinal symptoms, hypochondriasis, feelings of guilt, genital symptoms, general somatic symptoms, and anxiety. The third factor was comprised of the insomnia items (early insomnia, middle insomnia, and late insomnia) [32]. In our study, based on the highest representation of items, we also identified a three-dimension model: Dimension 1—psychic depression and motor retardation (depressed mood, work, activities, retardation, psychic anxiety, somatic symptoms: gastric, genital symptoms, and weight loss); dimension 2—disturbed thinking (guilt, suicidal thinking); and dimension 3—sleep disturbances and anxiety (early, middle, and late insomnia, agitation, somatic anxiety). In contrast to Wade’s findings on adult patients, in our study, guilt and suicide were loaded in a separate second factor.

Previous clinical descriptions of major depressive disorder in adolescents showed differences in symptom presentation compared to adult patients. It is especially emphasized that vegetative symptoms, such as appetite and weight changes, fatigue, and insomnia, may be common in adolescents with MDD [24,48,49] alongside somatic symptoms [50]. Rice et al. compared the clinical picture of depressive symptoms in adolescents and adults and showed that vegetative symptoms were more common in adolescent MDD, it being a separate symptom profile in depression for this age. They also underlined that a significant loss of energy was a core depressive symptom at an early age. Anhedonia, loss of interest, and problems with concentration were more commonly observed in adults [12]. Although these studies did not analyze factors using the HDRS scale, we observe some similarities with our results. It is possible that symptoms in adolescence, such as prolonged low self-esteem, feelings of guilt, loneliness, and suicidal thoughts with accompanying somatic and retardation symptoms, may influence or increase other depressive symptoms in this age. Previous studies indicate that, in adolescence, feelings of worthlessness and guilt are predictors of negative outcomes associated with depression in the future [51].

Studies comparing adolescent depression with adults also show the predominance of irritated mood in youths; this is why it is not surprising that a depressed mood rating in the HDRS may not appear to be a core symptom at this age.

In regards to anxiety factors, we did not confirm the previous results on adult patients, which showed that items related to both somatic and psychic anxiety were loaded in one common factor [47,52,53]. Our results align with other studies that identified two separate factors of somatic and psychic anxiety [33,46]. In the study by Pancheri et al. (2002), the gastro-intestinal item was loaded on two factors: somatic anxiety/somatization. Together with weight loss, they formed a separate factor 4—anorexia [54]. The insomnia items in adult samples were often extracted in a single factor [33,46,52,55,56]. In our analysis, insomnia was related to somatic anxiety, but the insomnia items, similarly to adult patients, had the highest loadings.

### 4.2. Correlations between the HDRS-17 Dimensions and BIS-11

The relationship between the impulsivity trait and depression among adolescents is still unclear because impulsivity has different facets. Attentional, motor, and non-planning impulsiveness were examined in this study. The BIS-11 scale was initially created for adult participants, but previous studies confirmed that it could be used in younger patient populations [57,58]. Earlier research on adolescent patients found significant and positive correlations between depressive symptoms and different impulsivity subtypes [37,38]. In our study, positive correlations between non-planning impulsivity and dimension 2 of the HDRS were observed, which consist mainly of disturbances in thinking as suicidality and guilt. Non-planning impulsivity could be defined as a “lack of future orientation” [45]. Previous studies indicated that non-planning impulsivity was correlated with decision-making processes in euthymic bipolar patients [59], and that this had a strong negative effect on adherence to medication in patients with mood disorders [60] as well as treatment dropout [61]. Poor self-esteem and interpersonal problems that are observed frequently in young patients seem to be more related to higher non-planning impulsivity and depression than to attentional and motor impulsivity subtypes [62]. Such results may indicate that depressive symptomology may influence impulsivity among younger patients via an inability to plan and a greater fixation on present thoughts and feelings, which may be a risk for suicidality. In contrast to the above, Khemakhem et al. (2017) found that impulsivity was not correlated with clinical features such as suicide attempts [63]. However, they compared 25 adolescents with MDD and 75 controls; a small study group could modify such results. Askénazy et al. (2003) reported that both impulsive and anxious young patients had more severe risk behaviors than the control sub-groups [64].

### 4.3. Correlations between the Dimensions of the HDRS-17 and TCI

Depressive symptoms and personality traits might be associated with various negative life outcomes during adolescence. In the correlation analysis of the HDRS factors with psychological factors such as personality traits, we found a positive correlation between harm avoidance and dimension 2 of the HDRS, consisting mainly of suicidal thoughts and guilt. The personality dimension of harm avoidance (HA) is characterized by excessive worrying, pessimism, shyness, and doubtfulness with a tendency to respond intensely to signals of aversive stimuli. Our findings are aligned with previous study results on temperament traits in young patients, which suggest that high harm avoidance is associated with a higher suicide risk and attempts in major depression [65,66,67,68].

Self-directedness (SD) is defined as the ability to regulate and control behavior to better adapt to a situation. It was shown that the SD dimension is associated with depressive symptoms and might be related to suicidal ideation [65]. In consensus with previous research, we observed a negative relationship between dimension 2 ‘disturbed thinking’ (suicidality and guilt) and self-directedness [69].

Previous research in unipolar and bipolar disorders shows that suicide attempts are associated with low reward dependence [67]. In a study of patients with a borderline personality disorder, Chapman et al. also reported that lower reward dependence is associated with higher suicidality [70]. Patients with low reward dependence scores are considered socially detached and indifferent [36,49]. In our study, reward dependence traits were negatively correlated with factor 2, which is in line with the observation on adult patients.

The most surprising result was the correlation with dimension 1 of the HDRS. Considering that, unlike other factors, it was the most heterogeneous, the obtained results should be approached with caution. In our study, we found a positive correlation between cooperativeness and dimension 1. Cooperativeness (C) reveals an overall individual sense of direction in one’s life and represents an individual’s character maturity, showing an inclination towards empathy, altruism, and respect for others. This is a somewhat unexpected finding in our study and might have a possible explanation in that these personality dimensions are often associated with the female gender. In our study, most patients were female, which could interfere with the results. Dimension 1 was also positively correlated with reward dependence.

The conceptualization of reward dependence as a construct has been characterized across distinct reward-related processes including motivation. According to Cloninger’s theory, individuals high in reward dependence are sociable, sensitive, sympathetic, and socially dependent, which may be disadvantageous in that patients are excessively socially dependent. High reward-dependent individuals also exhibit persistent behaviors and are easily influenced by emotions, which may be related to higher suicidality or self-aggression as commonly observed in adolescents who have less mature character [71,72].

## 5. Conclusions

Our study represents a first attempt to identify the HDRS-17 dimensions in young patients with depression and clinically understand the possible correlations between other psychological factors with the identified depression clusters. The three dimensions identified as core in adolescent and young patients with MDD were (1) psychic depression/motor retardation, (2) disturbed thinking, and (3) sleep disturbances/anxiety. When evaluating the correlations between the identified dimensions and psychological variables (impulsivity, personality traits), we found a positive correlation between the cooperativeness (C) temperament and dimension 1. Dimension 2 of the HDRS positively correlated with non-planning impulsivity and harm avoidance and negatively with self-directedness (SD). Dimension 3 of the HDRS was most positively correlated with reward dependence (RD).

These three identified depression clusters differ from those observed in the research on adult patients and seem specific for a young age. These differences are especially visible in the separate distinction of the HDRS-17 cluster relating to disturbed thinking (guilt, suicidal thinking). In adolescence, feelings of worthlessness and guilt might be risk factors and predictors of negative outcomes associated with depression in the future. In adolescence and young age, clinical observation confirms that decreased mood and depression, which predominates in adults, is often replaced by the irritated mood observed in youths. Sleep problems were mostly connected to anxiety, which may give more accurate treatment choice implications.

Our findings have some practical research implications. Identifying a separate symptom profile in youth depression may help in early intervention and a more accurate diagnosis at this age. Young patients with depression often experience various negative life outcomes during adolescence. Excessive worrying, pessimism, shyness, and doubtfulness make them less capable of regulating the negative emotions that they experience. Our findings confirm that high harm avoidance and low self-directedness, which influence adaptation and behavior, might be associated with a higher suicide risk and attempts in youths with major depression. Since impulsivity and specific personality traits are crucial psychological components that influence a patient’s reaction to stress and adaptation, the diagnosis of MDD in youths should involve different psychological components. The clinical interpretation of our findings shows that patients with mood disorders should be screened for personality traits and impulsivity to identify, as early as possible, those patients who are more likely to present a more severe course or worse outcome. Identifying reliable clinical and psychological predictors of therapeutic outcomes will allow for the development of a personalized approach that might individually tailor more specific interventions and evaluate novel therapeutic approaches in young patients

### Limitations

This study has limitations that must be taken into consideration when interpreting the results. First, we should consider our results preliminary, since our sample size was relatively small and included subjects from one geographical area, which might impact the generalization of our study findings. Another weakness is the low to moderate strength and power of the correlations. This study had more female than male participants, potentially raising concerns surrounding gender as a confounding factor. However, previous epidemiological research confirmed that females are more likely than males to develop MDD [73]. Lastly, the assessment of both personality traits and impulsivity were established using self-reported measures. However, the TCI and BIS-11 are among the most adopted instruments to assess personality and impulsivity features in clinical samples. In the future, our obtained results could be compared with available further studies. Thus, although promising, our results require further replication across young patients using a similar scale and further research into depressive dimensions, impulsivity, character, and temperament in depression in youths. Future studies involving longitudinal follow-ups are recommended, including a healthy matched-age control group. This will allow the role of personality and impulsivity as predisposing factors to specific MDD clusters or as resulting symptoms of depression to be more precisely distinguished.

## Figures and Tables

**Table 1:** Characteristics of the study sample.

Variable	Patients with Depression n = 52	
	N	%
Female	39	75
Race Caucasian	52	100
Inpatient	36	69
Drug free	21	40
Medication:		
SSRI	19	37
SNRI	3	6
TCA	2	4
other	7	14
Family history of any psychiatric disorder	37	71
Family history of affective disorder	29	56
	Mean	SD
Age (years)	18.67	3.54
Age at illness onset	16.82	2.96
Mean number of hospitalizations	1.25	0.80
Depression severity (HDRS-17 scores)	19.37	5.30

HDRS—Hamilton Depression Rating Scale, SSRI—selective serotonin reuptake inhibitors, SNRI—selective norepinephrine reuptake inhibitors, TCA—tricyclic antidepressants.

**Table 2:** Mean BIS-11 and TCI dimensions from the study group.

BIS-11 Scale		
	Mean	SD
Total	66.30	10.90
Attentional	17.99	3.16
Motor	22.80	4.48
Non-planning	25.50	5.23
**TCI Scale**		
	Mean	SD
Novelty seeking	20.04	6.45
Harm avoidance	24.65	7.12
Reward dependence	13.39	3.83
Persistence	4.04	2.01
Self-directedness	17.75	7.28
Cooperativeness	27.76	7.44
Self-transcendence	12.76	6.78

BIS-11—Barratt scale, TCI–Temperament and Character Inventory.

**Table 3:** Hamilton Depression Rating Scale (HDRS-17) principal component analysis.

Item #—Content	Dimension 1	Dimension 2	Dimension 3
1—depressed mood	0.625		
2—feelings of guilt		−0.680	
3—suicide		−0.682	
4—insomnia: early in the night			0.645
5—insomnia: middle of the night			0.734
6—insomnia: early hours of the morning			0.623
7—work and activities	0.582		
8—retardation	0.759		
9—agitation			0.632
10—anxiety psychic	0.433		
11—anxiety somatic			0.441
12—somatic symptoms gastro-intestinal		0.440	
13—general somatic symptoms	0.668		
14—genital symptoms	0.603		
15—hypochondriasis	-	-	-
16—loss of weight	0.553		
17—insight	-	-	-
Variance explained (%)	3.0	1.65	2.27

**Principal Component Analysis with Varimax rotation.** Factor 1: items: #1#7#8#10#13#14#16; Factor 2: items #2#3#12; Factor 3: #4#5#6#9#11; unassigned items: #15#17. Presented item loadings ≥ 0.4.

**Table 4:** Correlation of the HDRS dimensions (dimension 1, 2, 3) with clinical variables.

Variables	Dimension 1			Dimension 2			Dimension 3		
BIS-11									
	R	t	*p*	R	t	*p*	R	t	*p*
Attentional	−0.041	−0.294	0.769	0.014	0.100	0.920	0.007	0.054	0.956
Motor	−0.210	−1.520	0.134	0.027	0.193	0.847	−0.111	−0.792	0.431
Non-planning	−0.244	−1.780	0.081	0.310	2.310	**0.025**	−0.132	−0.943	0.349
Total	−0.188	−1.360	0.179	0.158	1.137	0.260	−0.078	−0.556	0.580
TCI									
	R	t	*p*	R	t	*p*	R	t	*p*
Novelty seeking	−0.168	−1.187	0.240	−0.196	−1.39	0.170	−0.117	−0.817	0.417
Harm avoidance	0.096	0.672	0.504	0.344	2.54	**0.014**	0.147	1.032	0.307
Reward dependence	0.288	2.086	**0.042**	−0.300	−2.18	0.033	0.314	2.297	**0.026**
Self-directedness	0.008	0.056	0.954	−0.357	−2.65	**0.010**	−0.061	−0.424	0.673
Cooperativeness	0.337	2.481	**0.016**	−0.089	−0.62	0.535	0.208	1.474	0.146
Self-transcendence	−0.157	−1.107	0.273	−0.102	−0.71	0.477	0.053	0.369	0.713
Persistence	0.186	1.315	0.194	−0.071	−0.49	0.622	0.180	1.272	0.209

Spearman’s correlation. TCI—Temperament and Character Inventory, BIS-11—Barratt impulsivity scale.

## Data Availability

Data is available on request.
